# An IoT and Fog Computing-Based Monitoring System for Cardiovascular Patients with Automatic ECG Classification Using Deep Neural Networks

**DOI:** 10.3390/s20247353

**Published:** 2020-12-21

**Authors:** Jaime A. Rincon, Solanye Guerra-Ojeda, Carlos Carrascosa, Vicente Julian

**Affiliations:** 1 Institut Valencià d’Investigació en Intel·ligència Artificial (VRAIN), Universitat Politècnica de València, 46022 València, Spain; jrincon@dsic.upv.es (J.A.R.); carrasco@dsic.upv.es (C.C.); 2 Department of Physiology, School of Medicine, Universitat de València, 46010 València, Spain; solanye.guerra@uv.es

**Keywords:** cardiovascular diseases, ECG, IoT, Fog-AI, LoRa, Edge-AI

## Abstract

Telemedicine and all types of monitoring systems have proven to be a useful and low-cost tool with a high level of applicability in cardiology. The objective of this work is to present an IoT-based monitoring system for cardiovascular patients. The system sends the ECG signal to a Fog layer service by using the LoRa communication protocol. Also, it includes an AI algorithm based on deep learning for the detection of Atrial Fibrillation and other heart rhythms. The automatic detection of arrhythmias can be complementary to the diagnosis made by the physician, achieving a better clinical vision that improves therapeutic decision making. The performance of the proposed system is evaluated on a dataset of 8.528 short single-lead ECG records using two merge MobileNet networks that classify data with an accuracy of 90% for atrial fibrillation.

## 1. Introduction

Cardiovascular diseases (CVD), such as high blood pressure, ischaemic heart disease or arrhythmias, are currently the leading cause of death in the world [1]. In Spain, 28.30% of deaths were related to CVD, according to the latest report published by the National Institute of Statistics (INE) on the causes of death in 2018 (https://www.ine.es/dynt3/inebase/es/index.htm?padre=6177&capsel=6179).

According to the European Society of Cardiology (ESC), “The clinical specialty of cardiology provides expert care for patients with heart and circulatory diseases” [2]. The cardiology service, one of the most demanded in the healthcare centers, has suffered very substantial changes caused by the current pandemic of COVID-19. For instance, in outpatient care, telematic consultation became very important at a time when it was necessary to suspend face-to-face appointments. To address this challenge, healthcare centers had to incorporate telemedicine systems, specifically telecardiology systems, which proved to be a useful and low-cost tool with a wide range of applications. In addition to providing support in the current pandemic situation, telecardiology supports those rural communities that are far from urban areas and lack specialized medical services.

In telecardiology systems, the primary diagnostic tool is the electrocardiogram (ECG), which represents the standard method for evaluating patients with cardiovascular disorders (rhythm or conduction disorders). [3]. In a conventional 12-lead ECG device, the electrical potentials of the heart are measured using 10 Ag/AgCl electrodes that are attached to different parts of the body surface [4]. Several monitoring systems are available on the market to record the ECG signal and send it to an analysis station. These systems include conventional devices for non-invasive monitoring such as the Holter or external cardiac event recorders [5]. However, monitoring systems have progressively introduced new devices for ECG capture that incorporate more comfortable and less intrusive sensors, known as wearable ECG monitoring systems [6,7]. Although now available, these systems were typically designed for recreational purposes and clinical experience remains limited. Therefore, there is still a demand for an easy-to-use, low-cost monitoring system that reduces diagnostic time and prevents the patient from travelling to the health center. In this case, the Internet of Things (IoT) could be the key to developing a wearable ECG monitoring system.

The IoT is a network of physical objects or devices that uses sensors and APIs to connect and exchange data over the Internet [8,9]. It makes it possible to share information in real time and also to collect and analyze data on a small and large scale, something that is already transforming the way medicine is organized and conceived. Currently, most interactions between a physician and a patient require equipment and devices, including external medical devices, such as glucose monitors; implanted, such as pacemakers; or stationary, such as home monitoring devices and scanners. Providing connectivity to these devices makes it possible to create an infrastructure of health systems and services: the internet of medical things (IoMT) [10]. The IoMT explosion is being driven by an increase in the number of connected medical devices that can generate, collect, analyze or transmit health data and connect to health care provider networks, transmitting or storing data in the cloud or internal servers.

The large-scale development of more efficient IoT and IoMT applications has been made possible thanks to the emergence of new hardware technologies such as radio frequency identification (RFID), wireless network technologies (Bluetooth, Wi-Fi, low energy ZigBee) and low energy wireless area network (LPWAN) technologies such as LoRa and SigFox, which help improve the connection of devices to the Internet.

Intelligent devices generate an enormous amount of IoT data that must be analyzed and exploited in real time, so artificial intelligence (AI) tools are required to process and give context to this data in order to generate actions without human intervention. AI algorithms can make more accurate and comprehensive diagnoses by offering personalized treatments. For instance, automatic ECG diagnosis has been studied for decades. In the early years, machine learning techniques were applied, such as fuzzy set theory [11], rough set theory [12], Hidden Markov models [13], artificial neural network [14] and Support Vector Machine [15]. However, the trend in automatic diagnosis points to the use of deep learning, which attempts to model high-level abstractions in data by using computer architectures that support multiple, iterative, non-linear transformations of data expressed in matrix or tensor form. This new automatic learning paradigm has opened the door to countless applications in the field of automatic diagnostic [16,17].

Many of these applications usually run on web servers that analyze the information sent by each of the devices. This massive sending of data causes a delay in the reception of the results, so the analysis can be done through a three-layer architecture. The first layer is the analysis embedded in the device, which is known as Edge Computing. The second layer is a Fog computing service that offers low latency and fast response time for healthcare applications [18].The third layer is the Cloud.

In this paper we propose a monitoring system for patients with cardiovascular diseases, specifically arrhythmias, equipped with an ECG device. The system is capable of sending the ECG signal to a service located in the Fog layer using the LoRa communication protocol. In addition, it includes a deep learning-based AI algorithm to help the physician make the diagnosis. This tool automatically classifies single short ECG lead records for the detection of Atrial Fibrillation and other heart rhythms. This monitoring system could be especially interesting for patients who live in rural areas or those who require telematic assistance during pandemics, such as the current COVID-19, since it allows the acquisition of bio-signals remotely and avoids the need for a face-to-face consultation.

## 2. State of the Art

ECG signal monitoring is a major concern in cardiovascular health care. In fact, many CVDs can be better diagnosed, controlled, and prevented through continuous monitoring systems. Currently, new technologies are being integrated into the development of ECG monitoring systems to provide efficient, cost-effective, fully connected and powerful systems. In this section we present the most relevant and related works that approach the same domain or characteristics, highlighting their contributions to the state of the art.

Holter-based ECG monitoring is the traditional monitoring system used in clinical practice. A Holter monitor is a 12-lead medical device connected to the patient’s body through electrodes that records heart rhythms continuously for 24 or 48 h [19]. The patient carries the Holter in a pocket or a bag placed around the neck or waist. After the established period for registration, the monitor is returned to the physician for analysis of the collected data. It is catalogued as a wearable and continuous monitoring device, since it enables the correct diagnosis of some CVD’s that may go unnoticed due to the absence of symptoms.

The current COVID-19 pandemic scenario has had an enormous impact on healthcare around the world, leading to a growing demand for remote health care that has accelerated the implementation of new monitoring systems to reduce the workload in a saturated healthcare system [20]. These new ECG monitoring systems are based on two types of emerging technologies: Monitoring devices and enabling technologies.

There are a great number of devices for ECG monitoring systems that can be classified as mobile devices, wearable devices and sensor devices, being the smartphone the most relevant device. For instance, Kailas et al. [21] developed a device that monitors heart activity, which is easy to operate and non-invasive. In addition, the power supply is supplemented by the energy harvesting from the mobile phone via the audio jack. It is also noteworthy that the incorporation of smart phones into monitoring systems has improved the acquisition and transmission of medical information. In this regard, Mahmud et al. [22] presented a prototype of a wireless health monitoring system to capture ECG and heart rate in real time using a smart phone case.

In ECG monitoring systems, enabling technologies support ECG processes, such as preprocessing, processing, storage, analysis and display of ECG signals. These technologies include IoT and Cloud/Fog computing.

Many of the studies related to IoT-based ECG monitoring systems evaluate the use IoT devices to capture ECG signals that are transmitted in real time over the Internet to the physician [23,24]. Nevertheless, other studies besides incorporating IoT devices, focus on improving data acquisition and processing. In this sense, Sundarasekar et al. [25] proposed the use of Maximal Overlap Discrete Wavelet Transform to decompose the ECG and identify changes in the R waves of the noisy signals. Similarly, Djelouat et al. [26] incorporated Compressive Sensing in an IoT-based ECG monitoring platform to leverage the ECG signal structure and achieve high efficiency in the acquisition. In addition, the platform provides an abnormality detection for each heart beat using different pattern recognition algorithms.

Many of these systems are not only used for monitoring elderly people, there are military applications that use these technologies in order to monitor soldiers in combat. Jethwa et al. [27] developed a system for monitoring the health of soldiers, which allows for the tracking of information from the war zone on the health status of each soldier. The system helps to improve the speed of decision-making and can prevent potential problems affecting soldiers’ health. Another important feature of the system is its low power consumption, due to the use of the LoRa module for data transmission instead of the high power consumption GSM/GPRS modules. There are also conceptual designs for an application in which the ECG will be monitored and transmitted via the LoRa to a remote location. Panagi et al. [28] mention that this is one of the most distinctive features that makes the LoRa an attractive technology for this application. It is the low implementation cost, the ease of implementation, the long area coverage and the acceptable transmission rate for the ECG. The authors identified several key components for the LoRa node that can be integrated into a small unit that will favour portability, scalability and ease of use. The authors focused on the development of the LoRa node and on performing functional tests with it to verify and characterize its performance.

Regarding Cloud Computing, some studies proposed the use of this infrastructure to provide storage and processing resources over the internet [29,30]. In this way, ECG signal processing is optimized and the cost of data transmission in ECG monitoring systems is reduced. Later, with the integration of monitoring systems to the cloud, and the use of Big-Data [31] as a tool for analysis and storage, it has been necessary to introduce intermediate stations in order to reduce high-power consumption. This can be improved by reducing the sending of data and processing it locally on the same devices, such as Fog devices.

Fog computing extends the cloud by migrating data processing closer to the production site, thereby accelerating the system’s responsiveness to events. The Fog infrastructure allows the management of multiple data from IoMT devices that send the information to the Fog node reducing the latency, response time or data delay [32,33,34]. Several low-cost applications using IoT and Fog devices can be found in the literature in order to streamline diagnostics. Gia et al. [35] proposed a Low Cost Health Monitoring (LCHM) model to gather the health information of different heart patients. The sensor nodes monitor and analyse the ECG signals to efficiently process the data of the cardiac patients, however the response time of the LCHM is longer which reduces the performance. Each of the sensor nodes acquires ECG, respiratory rate and body temperature, and then transmits this data to an intelligent gateway, which communicates wirelessly with the system to analyze the information and make an automatic decision. Mutlag et al. [36] developed a Multi-Agent Fog Computing (MAFC) model for healthcare critical tasks management, which significantly manages Fog computing resources by providing two levels of task prioritization (local and global). The MAFC model mapped between three decision tables to optimize the scheduling of critical tasks by assigning tasks with their priority, network load and network resource availability. He et al. [37] proposed a system to address the complexity and high number of personalized services in large-scale IoT-based healthcare applications. The framework, called FogCEPCare, used a set of custom services that work proactively using the complex event processing architecture of cloud computing. Bandopadhaya et al. [38] presented an integrated health monitoring system based on IoT and distributed computing for deployed soldiers. The proposed IoT architecture was service-oriented in three layers, where the computing functionalities were distributed among all the layers in order to improve the security of the soldier and to obtain a fast response to any unexpected event.

Cost reduction and communication efficiency are also an extremely important purpose of many monitoring systems. That is why some IoT-based monitoring systems include low-cost computer board such as the Arduino, as a signal acquisition tool, and the Raspberry Pi, as a Fog Computing tool. However, a new wireless transmission board has emerged on the market, the LoRa ESP-32, which allows data to be communicated over very long distances with low power consumption and could be used in health monitoring systems. In fact, some researchers have already proposed its use for monitoring people in adverse situations. For instance, Tayeh et al. [39] proposed a decentralized emergency alert system based on LoRa devices, which allow to automatically identify and locate a victim in areas without network coverage. The system consists of an intelligent clock that is used to obtain the user’s heart rate and physical activity, an IoT device with a GPS that transmits an alert through the LoRa device when the clock is activated, and finally a smartphone that displays the alert along with the victim’s location and address. This system was tested in a remote area located in the city of Belfort in France. Shobha et al. [40] presented a system based on the LoRa to track the movement of people in rural areas, forest areas and hiking sites. The authors highlight the low consumption of the battery, which makes it durable and suitable for long-term monitoring. They also point out the versatility of the LoRa, since it supports two-way communication, which is useful for rescuing people in emergency situations.

There is still a long way to go to achieve monitoring systems with complex computer requirements that are energy efficient and low cost. We aim to close the gap by presenting a prototype of a monitoring system for cardiovascular patients that includes new technologies such as IoT, Fog Computing, and deep learning to provide a cost effective, fully connected, and powerful ECG monitoring system.

## 3. Problem Description

For the next few years, in European countries, a progressive and important increase in cardiovascular pathology is expected due to the aging of the population, the increase in the presence of risk factors (smoking, obesity or sedentary life) and improvements in the quality of treatments with the consequent chronification of the disease [41]. For this reason, CVD continue to be a health priority that justifies and makes it essential to monitor them.

**Figure 1:**
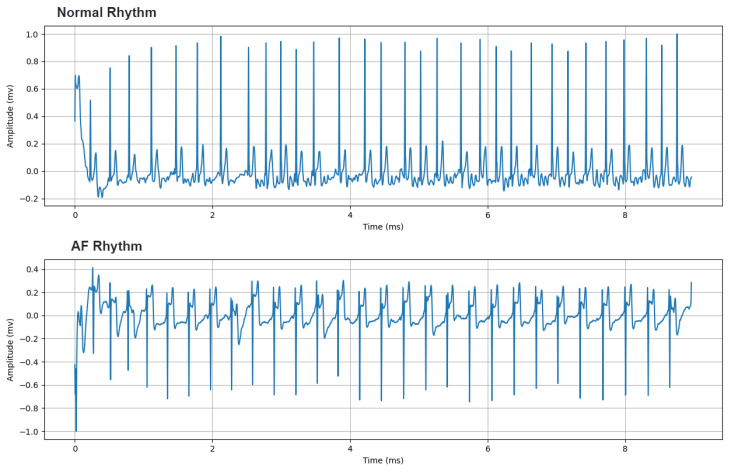
Samples of 1 lead electrocardiogram (ECG) records contained in the dataset of the 2017 Computing in Cardiology Challenge (https://www.physionet.org/content/challenge-2017/1.0.0/). From top to bottom, ECG with Normal Rhythm and Atrial Fibrillation.

Cardiac arrhythmias are one of the diseases most often treated by the clinical cardiologist. Atrial fibrillation (AF) is the most common cardiac rhythm disorder among the population and its incidence and prevalence are progressively increasing worldwide, especially in developed countries [42]. For instance, in European countries, it is estimated that 1–3% of the adult population is diagnosed with AF, exceeding 15% in people aged 80 or over [43]. While normal sinus rhythm, manifests as a single electrically activated wave front that propagates from the atria to the ventricles, AF is characterized by the presence of multiple errant wave fronts with different patterns of propagation that may be asymptomatic or result in symptoms such as palpitations, dyspnea and dizziness [42]. This causes characteristic elements to appear in the ECG signal such as discrete lack of P waves [44], as shown in Figure 1. Therefore, detection of AF can be problematic, even more when episodic occurrences of the arrhythmia are observed.

Consequently, the detection and treatment of this pathology requires continuous monitoring. However, the european health care model is oriented towards individualized attention, and is still not efficient for the high demand of chronic patients who make extensive use of health resources and need intensive and continuous monitoring. In addition, in the current pandemic situation, Nishiga et al. [45] report that chronic cardiovascular patients are at greater risk of suffering from COVID-19, having severe conditions with worse evolution and even death, so it is very necessary for them to follow the treatment of their disease through telematic consultation.

The monitoring of these patients optimizes and controls the fulfillment of treatments, thus preventing costly hospitalizations. Frequently, this follow-up takes place in the medical practice, although the current trend is to refer them to home care based on remote consultation through a telecardiology system [46], specially for those living in rural areas where access to primary or specialized medical services, such as cardiology, is often complicated by limited health, technical and human resources.

Telecardiology allows primary care health workers to interact in real or deferred time with cardiologists to avoid transfers and resolve emergencies. The primary care physician determines the need to conduct a telecardiology consultation for the patient based on the patient’s medical record, complete physical examination, and laboratory tests. If the patient agrees to deferred or real-time reporting, he or she will need to sign the informed consent form. In the medical office, the signals and images are acquired with the appropriate equipment and sent through a telecommunications network to the cardiologist. Once the cardiologist has received the information, it is displayed on a screen to be able to examine it and issue a diagnostic opinion, in order to suggest the most convenient specialized treatment. The technology used in a telecardiology consultation is a combination of portable diagnostic devices (e.g., ECG ), computer/intelligent phone and wireless communication infrastructure, which requires a stable data transmission network [46,47]. The transmitted data can be stored at a receiving station for immediate or later processing and examination. In cardiology, a wide range of invasive and non-invasive variables are used, and most of them can be recorded manually by the patients themselves (e.g., blood pressure, heart rate, three-lead ECG, body weight, or oxygen saturation).

**Figure 2:**
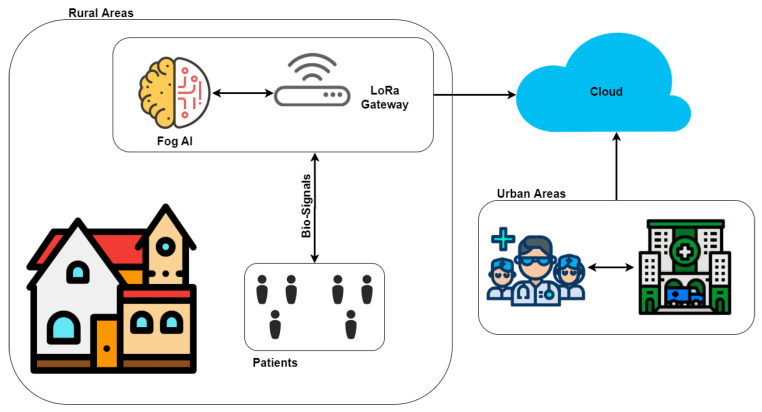
A general view of the problem description.

Currently, the telecardiology method proposed in the context of the remote ECG monitoring system is designed and developed for patients who do not necessarily require their presence at home. The trend in these systems is to have intelligent wearable devices capable of detecting situations of sudden falls, cardiac abnormalities, and hypertension/hypotension. therefore they are suitable for real-time monitoring, auto-diagnosis and remote diagnosis [48]. We believe that it is necessary that these type of systems should have the following features: (1) low cost and easy to use devices for the registration of bio-signals, especially the ECG; (2) automatic analysis tools supported by AI, that help the physician to diagnose, or notify the health center in case of a critical episode of the disease; (3) communications protocols that allow sending the information quickly and efficiently (Figure 2). In this way, it would be possible to intervene quickly with the activities carried out by the patient. All this thanks to the two-way communication that the system has.

## 4. System Architecture

**Figure 3:**
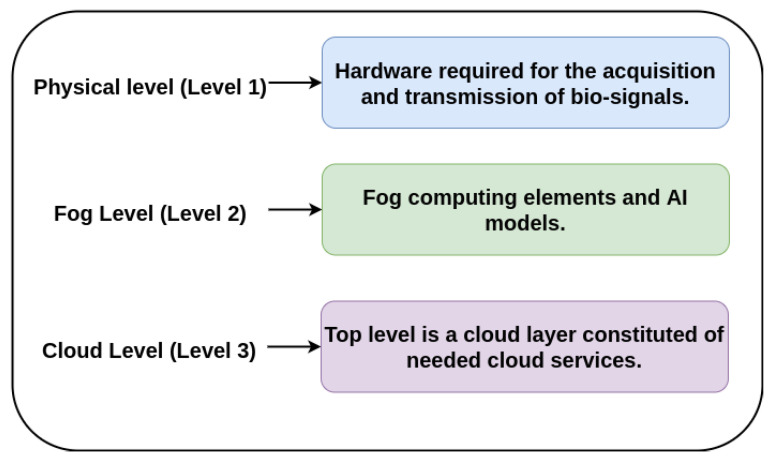
System architecture.

This section describes the architecture of the proposed monitoring system which has been structured in three levels as a typical generalized architecture of Fog computing [49,50,51] as can be seen in Figure 3.

1. Physical level (Level 1): formed of physical devices like the ECG device, in addition to other devices for capturing other bio-signals such as photoplethysmography (PPG), oxygen saturation (SpO2), phonocardiography (PCG) or temperature. These devices include an analog-to-digital conversion (ADC) system and a LoRa communication protocol for the transmission of the captured signals.
2. Fog Level (Level 2): is a middle level which consists of Fog computing elements typically called Fog nodes. In this case, these nodes includes software tools like deep learning algorithms and AI-Fog devices, which are responsible for analysing the ECG signal and also, the rest of signals, classifying them and making an auto-diagnosis.
3. Cloud Level (Level 3): The top level is a cloud layer constituted of needed cloud services or applications. In this case, this level involves health centers, hospitals and any related clinical service. These services receives alerts from the second level if some kind of cardiac anomaly is detected. If this is the case, the device sends the auto-diagnostic (pre-analysis) along with the data acquired, allowing the top level services to analyze the problem in depth.

The pillars of Fog architectures are security, scalability, openness, autonomy, reliability, agility, hierarchical organization and programmability. Moreover, the Fog Architecture defines the required infrastructure to enable building Fog as a Service (FaaS) to address certain classes of business challenges [51].

Fog architecture is a very promising technology that brings processing resources closer to the place where the data is generated, in our case the ECG acquisition, thus providing low latency and energy efficiency. This paper focuses on the description of the first two levels of the proposed architecture, since they constitute the core of the system. The information provided by these levels can be incorporated by services at the Cloud level provided by third parties and is outside the scope of this paper. According to this, the following sections will describe in detail the physical and Fog levels.

### 4.1. Physical Level

The physical layer actually consists of a set of capture devices to gather data on health-related signals to diagnose cardiovascular diseases. The data collected is then transmitted to the center fog layer to make the processing and prediction in real time. According to this, this section mainly describes the proposed equipment for capturing, pre-processing, transmission and analysis of bio-signals, specially the ECG.

**Figure 4:**
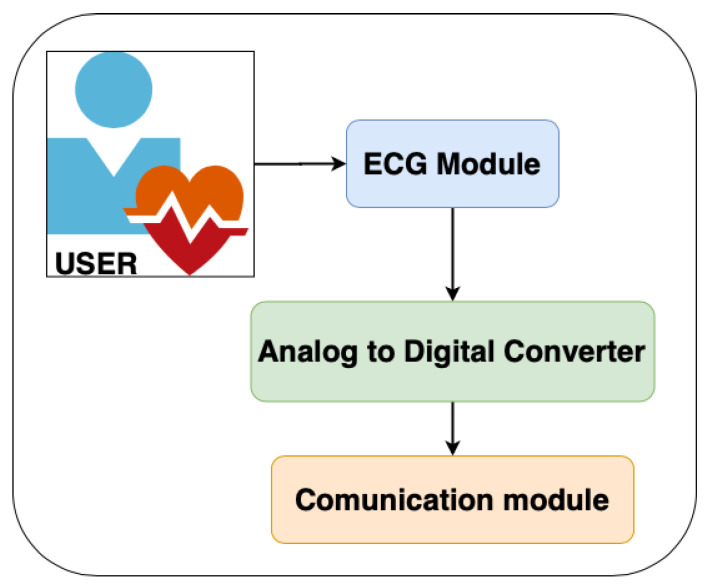
Architecture of Level 1.

**Figure 5:**
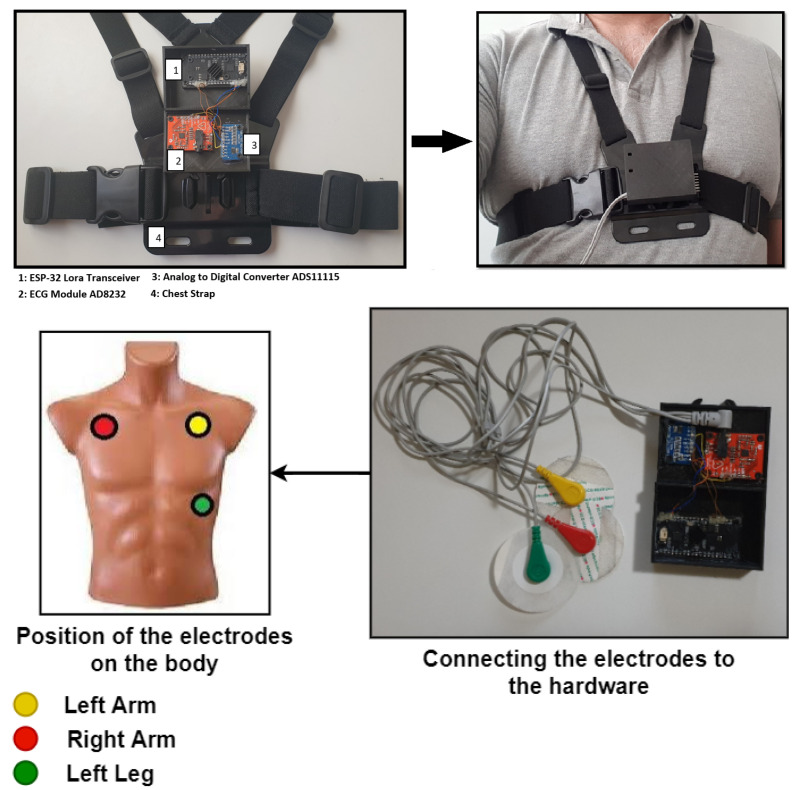
Hardware Deployment.

The layer for capturing and pre-processing the ECG signal is divided into three stages. The first stage is responsible for capturing the ECG. This stage integrates a differential amplifier instrument AD8232, which is responsible for amplifying the heart signal by raising the amplitude from milli-volts (mV) to approximately 3.3 volts. The second stage includes an analog-to-digital converter ADS1115 that transforms the analog signal coming from the AD8232 to its corresponding digital value (with a 16-bit resolution) (Figure 4 shows the proposed architecture while Figure 5 shows how the hardware is deployed).

**Figure 6:**
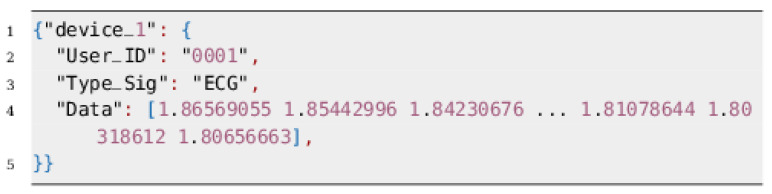
The data structure employed by ESP-32 to send the signal.

In the stage three, the digital value corresponding to the amplified analog signal is transmitted to a ESP-32 board via an I2C protocol. This board is responsible for receiving the ECG data and transmitting it to the Fog device using a built-in SX1276 LoRa chip. The data structure with LoRa protocol is shown in the Figure 6.

LoRa is a LPWAN protocol integrated into devices with limited power (e.g., battery powered) and transmission of a few bytes at a time. LPWAN technologies have been developed to enable new human-centered wireless and health monitoring applications [52]. In these devices the data traffic can be initiated by the end-user device or by an external entity that wants to communicate with the end-user device [53,54]. LPWAN and its open protocol LoRaWAN^®^ has become the solution for many applications, featuring some of 5G’s strengths that allow it to take advantage of an open standard and a thriving global ecosystem. A LoRa device has high range of communication that can easily reach more than 10 km, being greater than that offered by Bluetooth^®^, Wi-Fi or the 5G mm. Also, it operates in the 900 MHz ISM (Industrial Scientific and Medical) band with a data rate between 0.3–50 Kb/s. Although the data rate could be acceptable for the transmission of signals such as ECG [55], we do not use real-time data transmission in our system. In this sense, the data is stored for a period of time (1 min approx) and then transmits it to the Fog module for classification.

The data structure used in sending the ECG to the Fog node converts the signal into an image. This image is analyzed by the second level, in order to determine if the user has any cardiac anomaly. This will be explained in next section.

### 4.2. Fog Level

This second level is responsible for handling all incoming data generated on the first level and processing it to determine if there is an arrhythmia in the ECG recording.

The Hardware part of this level is formed by a low-cost raspberry pi system. This system will be in charge of the ECG data classification process.

**Figure 7:**
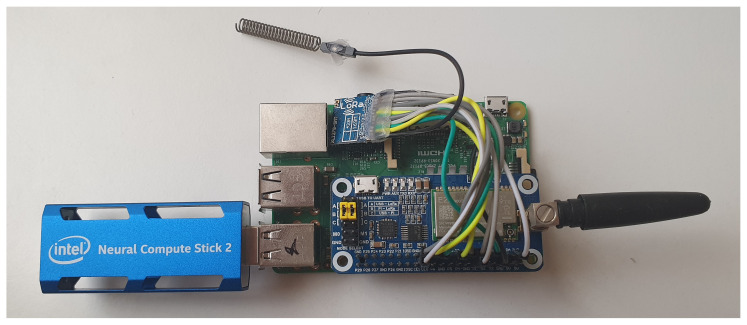
Fog-artificial intelligence (AI) System.

However, this system does not have the necessary computing power to execute deep learning models, so it was necessary to incorporate a co-processor, such as Intel Neural Compute Stick 2 (NCS 2) (Figure 7), which decreases the classification time. The Intel NCS 2 is aimed at cases where neural networks must be implemented without a connection to cloud-based computing resources. It offers quick and easy access to deep-learning capabilities, with high performance and low power for integrated IoT applications, and affordably accelerates applications based on MobileNet [56] and computer vision.

At the end of the process, the raspberry pi system is connected to a WAN network, which can be wired, 4G or 5G. In this case, we used a 4G USB modem, which allows the system to send the information obtained from the patient to a hospital, clinic or health center, to be analyzed by the doctor. The use of a wireless system for sending data packages gives the system the ability to be placed anywhere in the city.

For the classification process we used a MobileNet network, a computer vision model for the open source platform TensorFlow. MobileNets are small, low-latency, low-power models that can be used for classification, detection, embeddings and segmentation processes. These models were designed to operate quickly with high accuracy in a resource-constrained environment, such as a device or an integrated application. For our system, we designed two MobileNets which were trained in the cloud and later embedded in the raspberry pi.

For the classification, it was considered to group the ECG signals into four classes of rhythm patterns: *Normal sinus rhythm (Nsr)*, *Atrial fibrillation (Af)*, *Other rhythm (Or)* and *Too noisy to classify (No)*, based on the data set of the 2017 Computing in Cardiology Challenge (https://www.physionet.org/content/challenge-2017/1.0.0/). The data set contains 8528 short (9–60 s long) and single-lead ECG recordings donated by AliveCor. ECG recordings were acquired and band pass filtered by the Kardia device Mobile™. All data were provided in MATLAB V4 WFDB-compliant format.

The extraction of features that determine the class of each signal was done by applying analysis in the time and frequency domain.

### 4.3. Time Domain Signal Analysis

**Figure 8:**
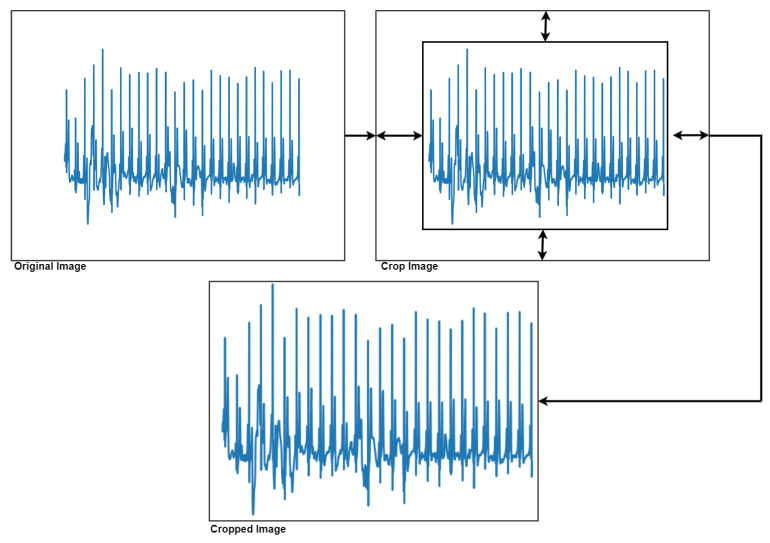
Extraction of the area of interest from the image.

To achieve this classification process, it was decided to convert the ECG signals into images. First, it was necessary to perform a series of image pre-processing steps to remove all the axis in the image, as well as crop the image by extracting the area of interest (Figure 8).

**Figure 9:**
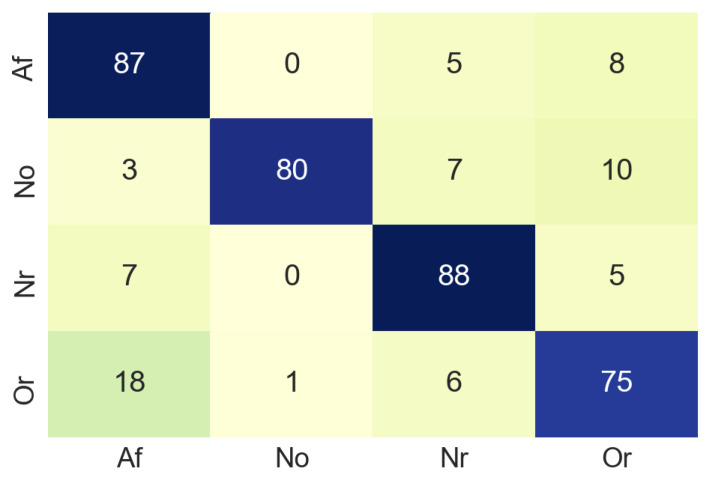
Confusion matrix of time domain.

The next step was to resize the image from 1500 × 600 to 224 × 224 pixels, since to train the MobileNet is necessary that all the images have the same size (224 × 224). The designed MobileNet has the following hyperparameters: Epochs: 70, batch size: 512, learning rate: 0.001. In the training process, the test accuracy was about 0.6 due to the limited amount of dataset samples. The MobileNet was validated by using a confusion matrix (see Figure 9) built from 100 non-used images from the original dataset. In the confusion matrix it can be seen that in most cases the classification is done correctly, although it should be noted that 18% of the images belonging to the class Or, have been classified as Af. This is a slightly elevated value for a false negative, the normal for this case is that the value should be close to 0. Table 1 summarises the accuracy values of the classification process in the time domain. For class Af the accuracy is 87%, which suggests that our model works well enough in the classification process.

### 4.4. Analysis of Signals in the Frequency Domain

**Figure 10:**
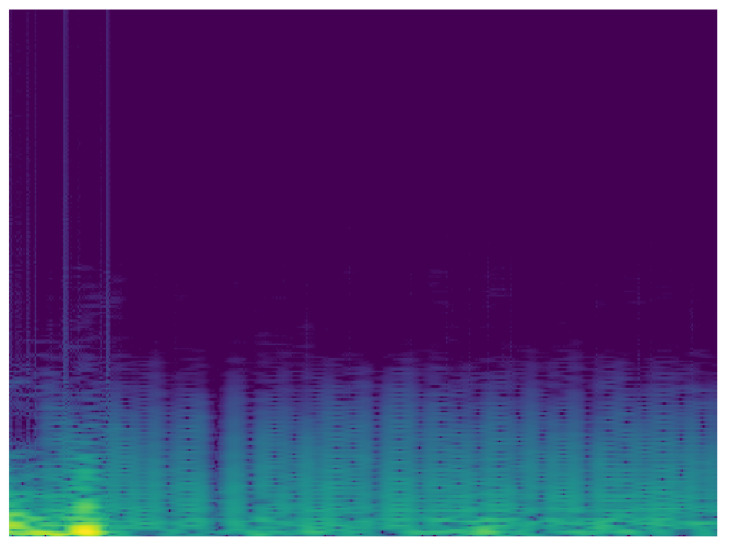
Spectrum of ECG signal with normal rhythm.

**Figure 11:**
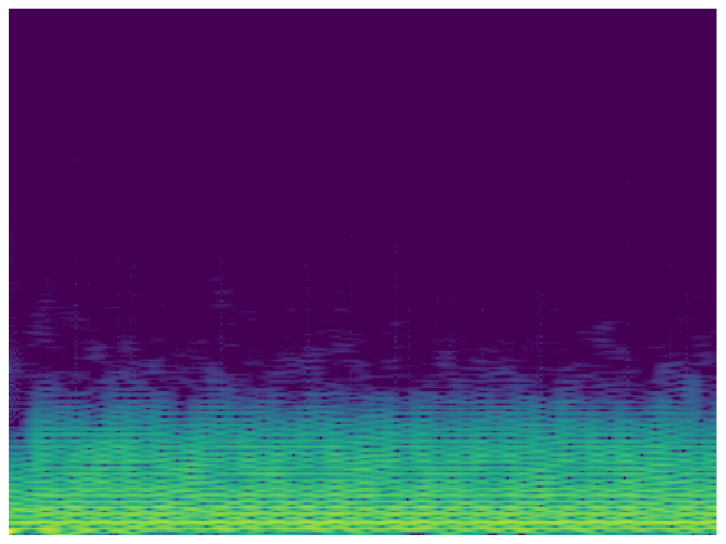
Spectrum of ECG signal with atrial fibrillation.

Taking into account the possibility of improving the classification, it is proposed to carry out the analysis of the signals in the frequency domain. In this sense, another MobileNet network was built with the same characteristics of the analysis in time. To analyze the ECG signal in the frequency domain, the spectrum of the frequency components was used, showing how the signal’s energy is distributed over a range of frequencies. Figure 10 and Figure 11 show the frequency content of an ECG signal with normal rhythm and with atrial fibrillation respectively.

**Figure 12:**
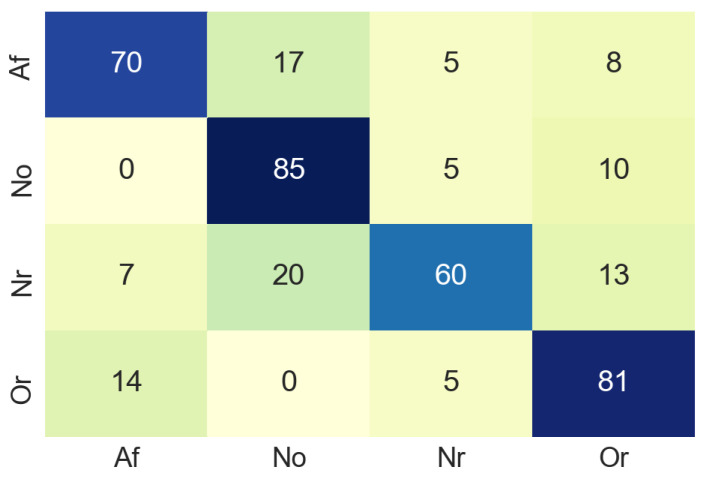
Confusion matrix of frequency domain.

The same pre-processing steps for time domain images were performed for the classification with spectrum images. As a result of this classification process, the confusion matrix was extracted using the data for validation. The confusion matrix obtained using 81 non-used images from the original dataset, the result of this process can be seen in the Figure 12. In this case, in the confusion matrix obtained in the classification process using the spectrogram images, it can be observed that in 20% of the cases the system classifies the input as class No when it should be class Nr. This value is excessively high, although the values of the correct classifications are more than acceptable.

Nevertheless, Table 2 summarises the accuracy values of the classification process in the frequency domain. For class Af the accuracy values was 70%. In this case, a lower accuracy value for class Af may be due to the color uniformity of the images. However, it is a good classification result and an indicator of the correct performance of the proposed model.

Considering that two MobileNet networks have been used to analyze two types of images, the result obtained from the classifications is considered acceptable. In both cases we obtained percentages of classification that did not go below 60% of accuracy. The next step was to merge these two networks and from this union the real class associated to the input image was extracted. This process is explained in the following subsection.

### 4.5. Merging Process of the Two Classifications

**Figure 13:**
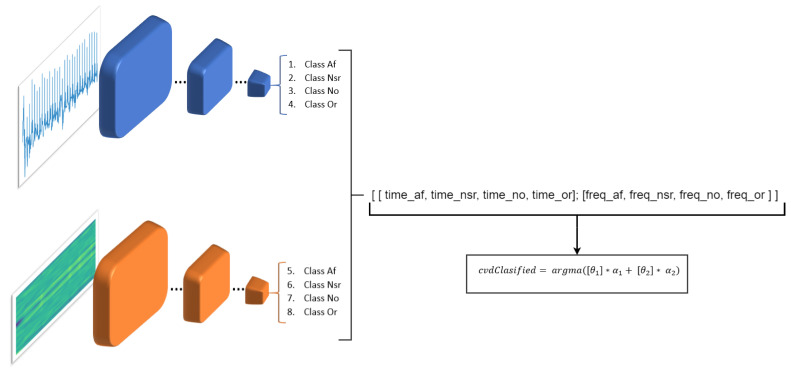
Merging process of the two classifiers.

The result of these two networks makes it possible to have a better classification, which would be similar to having the opinion of two different doctors who analyze the same signal with different methods. Consequently, we merge the output of the two MobileNets, the one that analyzes the signals in the time domain (MobileNet-1) and the other that analyzes the signals in the frequency domain (MobileNet-2). This is done in order to obtain a more solid decision about which is the real class. To obtain this output we use the following equation:

(1) $$cvd\_classified=argmax(\left[\theta_{1}\right]*\alpha_{1}+\left[\theta_{2}\right]*\alpha_{2})$$

where cvd_classified denotes the final result of the classification. $\alpha_{1}$ is the output that analyzes the signals in the time domain and is a probability vector in which the probability of being one of the four classes is expressed. $\alpha_{2}$ is also a probabilistic vector but represents the output of the MobileNet-2 which performs the classification in the frequency domain. $\theta_{1}$ and $\theta_{2}$ are the weights for the MobileNet-1 and MobileNet-2 classifiers respectively (Figure 13).

For the merging process, the dataset was divided into three parts, following the distribution standards when using neural networks: 80% for training, 10% for testing and 10% for validation.

**Figure 14:**
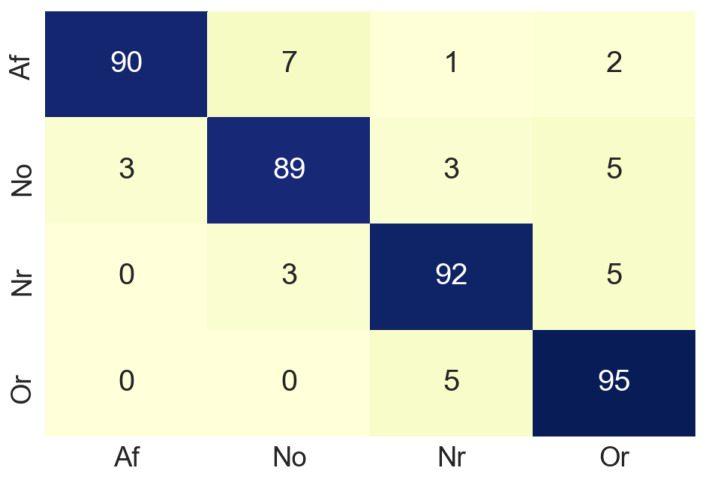
Confusion matrix of merge process.

Figure 14 shows the confusion matrix obtained from the merging process and Table 3 summarizes the accuracy values of the merging process. For class Af the accuracy values was 90%. This allows us to infer that the combination of the results obtained when analyzing the signals in time and frequency, gives better results than when analyzing the signals separately.

To illustrate the classification process with a real case, a healthy patient’s ECG (without cardiac arrhythmia) was recorded for one minute. The ECG was captured over an entire day using the device described in this document. The signals were captured at intervals of 5 min each. Accordingly, about 200 images were obtained during the testing process. Once the signals were captured and sent to the AI-Fog device, this transmission process took approximately 1 min and 3 s.

**Figure 15:**
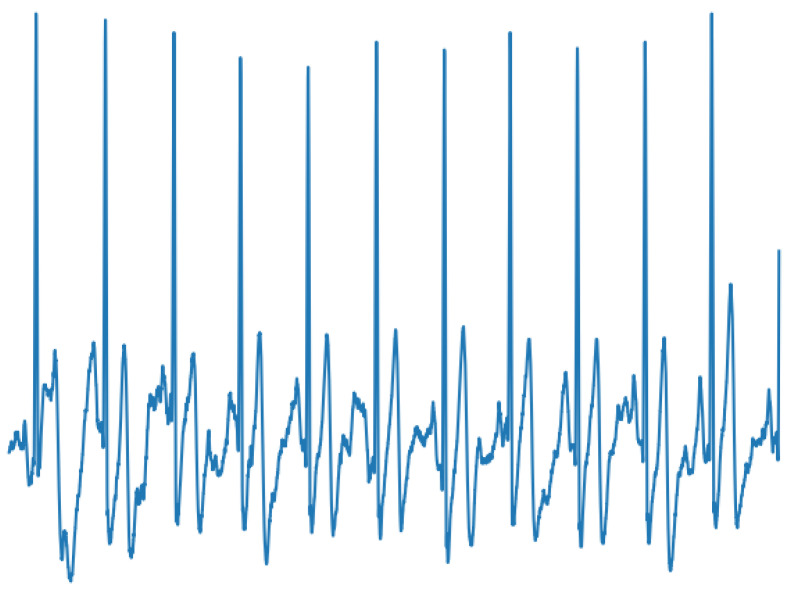
Pre-analysis of the signal was: Normal Sinus Rhythm. Waiting time for classification: 19 ms. (No magnitudes are indicated to avoid noise to the neural network).

Later, the conversion of the images was performed, using the same pre-processing of the training phase. The images were resized to (224 × 224) and the axes and magnitude values were eliminated to avoid noise that interferes with classification process. Figure 15 shows an ECG signal obtained from a healthy subject using the proposed monitoring system. In the generated input signal shown in Figure 15, the system determined that the user had a Normal Sinus Rhythm, and the time it took the system to perform this classification was 19 ms.

The results of the merged model indicate that this model seems to work correctly with all the images tested, allowing the correct classification of the classes that were previously trained.

## 5. Conclusions and Future Work

The world is facing an alarming growth in cardiovascular disease due in part to an unhealthy lifestyle. Thanks to the high level of sophistication that new technologies have reached in areas such as informatics and telecommunications (e.g., IoT devices or AI techniques), effective solutions can be developed to address this challenge. In this sense, we present a ECG monitoring system for cardiovascular patients. The system incorporate a deep learning tool based on fog computing in order to perform an early detection of Atrial Fibrillation and other heart rhythms.

The proposed approach was validated with a small group of elderly people from a day care center near the city of Valencia (Spain). In this center, the necessary infrastructure was installed to simulate a remote monitoring situation. Preliminary results indicated that the system is capable of identifying abnormal heart rhythms. Similarly, the results obtained were evaluated by medical personnel without any problems being detected in the use of the system.

Compared to traditional methods for signal analysis, the proposed method analyzes the ECG in the time and frequency domain, obtaining two classification models that when merged improve the accuracy of the procedure. Once the tests were completed, the model was incorporated into the Fog system, which included an Intel NCS2 processor that optimizes the classification and speeds up the process. It also incorporates a LoRa communication system, that compared to other transmission methods such as Bluetooth or Zigbee, has a longer range and low power consumption. This makes it ideal for the design of portable devices. Once the network has been trained and the system sends the ECG, the classification result can be obtained quickly.

Future work will initially focus on acquiring other bio-signals such as photoplethysmography, phonocardiography or oxygen saturation. This will allow us to have other variables that increase the quality of monitoring the cardiovascular patient and advise the specialized medical staff. At the same time, we will continue, as far as possible, to carry out more tests, collaborating with a greater number of elderly people and thus expanding the experiments carried out to date.

## Figures and Tables

**Table 1:** Accuracy for each class in time domain. Samples used are the same ECG signals converted into images.

Class	Accuracy (%)	Number of Samples
Atrial fibrillation (Af)	87	100
Normal sinus rhythm (Nsr)	80	100
Too noisy to classify (No)	88	100
Other rhythm (Or)	75	100

**Table 2:** Accuracy for each class in frequency domain.Samples used are the same ECG signals converted into images.

Class	Accuracy (%)	Number of Samples
Atrial fibrillation (Af)	70	100
Normal sinus rhythm (Nsr)	85	100
Too noisy to classify (No)	60	100
Other rhythm (Or)	81	100

**Table 3:** Accuracy for each class in merge process. Samples used are the same ECG signals converted into images.

Class	Accuracy (%)	Number of Samples
Atrial fibrillation (Af)	90	100
Normal sinus rhythm (Nsr)	89	100
Too noisy to classify (No)	92	100
Other rhythm (Or)	95	100

## Abbreviations

The following abbreviations are used in this manuscript:

CVD	Cardiovascular diseases
ECG	Electrocardiogram
AF	Atrial fibrillation
IoT	Internet of things
IoMT	Internet of Medical Thins
LPWAN	Low energy wireless area network
NCS2	Neural Compute Stick 2
